# Hairy tongue

**DOI:** 10.1503/cmaj.201559

**Published:** 2021-04-19

**Authors:** Eric Burge, Siddharth Kogilwaimath

**Affiliations:** College of Medicine (Burge); Division of Infectious Diseases, Department of Medicine (Kogilwaimath), University of Saskatchewan, Saskatoon, Sask.

**Figure 1:**
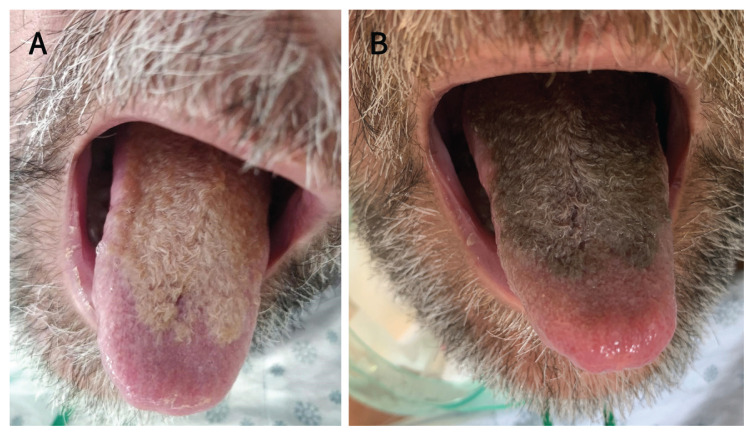
Photographs from a 55-year old man with hairy tongue. (A) Hair-like coating on the dorsum of the tongue after 1 month in intensive care. (B) Four days later, the colour of the coating changed markedly after the patient transitioned from tube feeds to oral intake, and from fluconazole to ciprofloxacin.

A 55-year-old man developed a new, hair-like coating on his tongue after a month in the intensive care unit (ICU) with Guillain-Barré syndrome. He had been intubated for 11 days and had had a tracheostomy. Aside from distress about the appearance of his tongue, he was concerned about a decreased sense of taste. The patient had no other oral complaints. He had a 30 pack-year history of smoking. During his stay in the ICU, he received piperacillin-tazobactam, trimethoprim-sulfamethoxazole, ciprofloxacin and quetiapine. The patient’s management team initially diagnosed oral candidiasis and treated him with several courses of oral nystatin and systemic fluconazole. Non-response to antifungal medications, combined with a typical clinical appearance and history, led us to diagnose hairy tongue (Figure 1).

Hairy tongue is a benign condition resulting from elongation of the filiform papillae because of keratin build up. This can result from inadequate exfoliation (e.g., from decreased oral intake, poor oral hygiene or dry mouth related to anticholinergic drugs) and accelerated keratinization (as may occur with irritation from smoking).^1^ Hairy tongue appears as a hairy coating on the tongue’s dorsum, sparing the tip and lateral borders.^2^ The colour ranges from cream to brown to black, depending on extrinsic factors, such as diet and smoking, and intrinsic factors, such as chromogenic bacteria and fungi).^1^,^3^

Hairy tongue is usually asymptomatic. While the diagnosis is clinical, tongue biopsy may rarely be required to clarify the diagnosis if the lesion does not respond to conservative management. 1 As with our patient, hairy tongue is frequently confused with pseudomembranous candidiasis and treated ineffectively with antifungal therapy.^3^ The management of hairy tongue consists primarily of gentle débridement with a soft-bristled brush along with reassurance.^1^ Aggravating agents should be discontinued, and risk factors modified where possible. Hairy tongue often resolves spontaneously.

We think our patient’s hairy tongue was caused by a prolonged period of limited oral intake, xerostomia related to the anticholinergic effects of quetiapine and a change in oral flora from various courses of antimicrobials. The patient’s tongue improved substantially after 2 months.
